# Training community health nurses to measure parent–child interaction: a mixed-methods study

**DOI:** 10.1093/eurpub/ckz155

**Published:** 2019-09-20

**Authors:** Penny Levickis, Cristina McKean, Elaine Walls, James Law

**Affiliations:** 1 Melbourne Graduate School of Education, University of Melbourne, Melbourne, Australia; 2 Clinical Sciences, Murdoch Children’s Research Institute, Melbourne, Australia; 3 School of Education, Communication & Language Sciences, Newcastle University, Newcastle upon Tyne, UK; 4 Nursing, Midwifery & Health, Northumbria University, Newcastle upon Tyne, UK

## Abstract

**Background:**

This study aims to determine whether the Parental Responsiveness Rating Scale (PaRRiS) completed at child age 24–30 months can be used by community child health nurses (CCHNs) to reliably measure the quality of parent–child interactions in practice.

**Methods:**

A mixed-methods design was used involving CCHNs working in public health settings. Five CCHNs recruited from the North-East of England were trained to use PaRRiS. Thirty parent–child dyads attending their routine 24–30-month check were observed. Nurses rated parent–child dyads during 5 min of free-play using PaRRiS. The free-play sessions were video recorded and rated blind by the first author to the nurse observation. Semi-structured phone interviews were conducted with the five CCHNs once observations of parent–child interactions were complete. Interviews were audio-recorded, transcribed, anonymized and thematically analyzed.

**Results:**

Two-thirds of participating parents were mothers. Half the families (15/30) were from the 10% most deprived areas based on the English Index of Multiple Deprivation. The average PaRRiS score was 3.03 [standard deviation (SD) = 0.8; all ratings were <5.0]. Reliability between the first author (‘gold standard’) and CCHNs was excellent [Intra-class correlation coefficient (ICC): 0.85; 95% confidence interval (CI): 0.67–0.93]. CCHNs found PaRRiS aligned well with current practice and was acceptable to parents. There was no evidence of a relationship between social disadvantage and PaRRiS scores.

**Conclusions:**

With further development and evaluation work, PaRRiS could potentially be incorporated into existing universal health services to provide child health nurses with an additional tool for identifying families most likely to be in need of parent–child interaction interventions.

## Introduction

Acquiring good oral language skills (i.e. using and understanding language effectively), is vital to both individual well-being and human health. The first 5 years of life are a critical period for developing these skills and while many children appear to acquire language with ease, one in five children under five will struggle to develop adequate oral language.^1^^,^^2^ Prevalence studies^3^^,^^4^ suggest 5.8 million children and adolescents across Europe are affected by language difficulties placing them at risk of lifelong deficits in communication, academic achievement, social-emotional well-being, employment opportunities and health literacy.^5–7^ The prevalence and clear social gradient of language difficulties has led to recent calls for public health prevention approaches to addressing child language difficulties and social inequalities.^8^^,^^9^

Health, education and care policies around the world identify the ‘early years’ (0–5) in childhood as vital for promoting robust life-course development and reducing inequalities. In the European Region recent policy identified non-communicable diseases and developmental disorders, such as language difficulties, as emerging major societal challenges.^10^ One of the most commonly observed phenomena in cases of language difficulties is restricted parent–child interaction, that is, the amount and quality of parent–child interaction are related to a child’s development of language skills.^11^^,^^12^ Specifically parenting that is contingent, developmentally appropriate and prompt in response to a child’s initiations is posited to promote greatest language growth.^13^

There is a strong association between social disadvantage and language difficulties, with the distribution of language abilities following a clear social gradient.^14^ How well or poorly a child acquires language is widely acknowledged to be determined by an interplay between genetics and environmental factors^15^ with the early language environment being one factor thought to partially mediate the relationship between socioeconomic disadvantage and oral language skills. In particular, parental linguistic input is shown to be associated with parent education level, with more educated parents providing a richer language environment.^16–18^ Recognition of the importance of quality parent–child interaction in promoting language development has resulted in the implementation of parent-focused language interventions. Such interventions may target socioeconomically disadvantaged groups (targeted selective approach) or children with identifiable low-language abilities (targeted indicated approach).^19–21^ However, there is limited evidence supporting the effectiveness of these interventions as a preventative public health intervention to improve child language outcomes and reduce inequalities.^3^ Crucially, children in control arms of such studies make equivalent positive progress as those receiving parent–child interaction interventions.^22^ One possible reason for these null findings may be inappropriate targeting. It may be that some of the parents recruited to these studies are already highly responsive to their child and providing quality input.

It has been suggested that identifying children at risk through the integration of child and family factors, including measures of parent–child interaction may result in more accurate intervention targeting.^23^ Measures of parent–child interaction shown to be associated with child language outcomes^24^ could assist in identifying children most likely to benefit from parent–child interaction interventions, but detailed ratings of parent–child interaction are typically time-consuming and costly.^25^ A global rating tool may be a more efficient, accessible method for measuring quality of parent–child interaction. Preliminary work was carried out by the first author and colleagues^26^ in developing an observational rating scale of parent–child interaction, specifically, a measure of parental responsiveness (PaRRiS: Parental Responsiveness Rating Scale). In this work, video clips of mother–toddler dyads during free-play were rated blind on the PaRRiS tool at age 2 years and language outcomes were assessed using standardized language measures (mean: 100; SD 15) at ages 3 and 4 years. In adjusted linear regression models [potential confounders: gender, maternal education and socioeconomic status (SES)] PaRRiS ratings strongly predicted receptive, expressive and total language standard scores at age 3 (coefficient = 5.9, 5.4 and 6.2, respectively; *P *< 0.001 for all) and 4 years (coefficient = 4.6, 3.1 and 4.0, respectively; *P *< 0.001 for all).^26^ A high level of inter-rater reliability was achieved for PaRRiS with Cohen’s kappa of 0.79 (84.6%).^26^ Findings showed that it is feasible to train speech and language therapy students to use PaRRiS efficiently and reliably in a large community-based sample of mother–child dyads, and toddlers of mothers with higher PaRRiS scores had higher language scores at ages 3 and 4 years. However, if this measure is to be implemented in public health practice it is essential for both reliability and acceptability to be found with the professional group most likely to use it—namely community child health nurses (CCHNs).

Therefore, the aim of this study was to determine the acceptability, reliability and feasibility of training UK CCHNs (health visitors and nursery nurses in the UK) to use PaRRiS during routine child health checks as a measure of parental responsiveness. Specifically, study questions were:

1) Can child health nurses reliably rate live a 5-min observation of parent–child interaction during routine practice using the PaRRiS tool?
2) What are child health nurses’ views and experiences of PaRRiS training and the feasibility and acceptability of its application in practice?

## Methods

### Study design and setting

The study took place in the North of England (July 2017–September 2018). CCHNs, that is health visitors and nursery nurses from a National Health Service Trust, were invited to participate in this study. Health visitors are registered nurses/midwifes with additional training in community public health nursing. In the UK, health visitors work with parents of newborns, offering support and advice from the ante-natal period until school entry at age 5 years. Health visitors often work with community nursery nurses who support health visitors’ practice and who have qualifications in childcare. Thus, both nursery nurses and health visitors were eligible to participate.

The first author (P.L.) attended a health visitor service meeting to share study information, answer questions and offer invitations to participate in the study. Health visitors and nursery nurses were asked to indicate their interest to the health visitor lead who passed their contact details to the study team. During the initial phase of recruitment, one health visitor and two nursery nurses were recruited and trained. Due to difficulties in room availability and recruitment of parent–child dyads taking longer than anticipated a second phase of recruitment was conducted and two additional nursery nurses recruited and trained. In total, one health visitor and four nursery nurses participated in the study.

### PaRRiS training of CCHNs

CCHNs attended a 1-h workshop with the first author. They were given an overview of the background and development of the rating scale, a copy of the publication detailing the rating scale,^26^ and were shown five examples of parent–child dyads during free-play. P.L. had pre-rated the example clips and the nurses assigned ratings to then compare with P.L.’s ratings. Nurses were given an additional three example videos to take away and rate in their own time. They then emailed their ratings to P.L. who compared them with her own and provided feedback, with CCHNs needing to achieve a minimum of 80% agreement.

### Recruitment of parents and their toddlers

The trained CCHNs invited caregivers whose child was due for their 24–30-month health review to take part (November 2017–July 2018). Only families attending a centre for their review, not those having their review at home, were eligible to participate. For logistical reasons, participating nurses had identified a preference for the study setting to be in centres rather than in family homes. It was made clear to parents that study participation was voluntary and would not affect their usual care. Interested parents were provided with an information statement and consent form. P.L. was available to go through the consent form verbally to ensure participants understood what was being asked of them. Of the 31 parents invited to take part (i.e. all parents attending one of the participating centres for their review during recruitment), 30 (96.8%) consented and completed the parent–child free-play session. The parent who did not consent to take part was invited at the end of the review and they did not have the time to complete the free-play session. Of the 30 families taking part, 28/30 (93.3%) provided complete data (observation and questionnaire), with missing questionnaire data for two participants.

All participant CCHNs and parents gave written informed consent. The study received ethical approval from the National Health Services East Midlands—Nottingham 2 Research Ethics Committee (17/EM/0088) and Health Research Authority approval (project ID: 208243). All CCHN’s names were changed when interviews were transcribed for purposes of anonymity.

### Parent–child interaction procedures

The free-play sessions were conducted either at a children’s centre or healthcare centres. For those parents who consented to participate, the trained CCHNs observed 5 min of parent–child free-play and rated the interactions using PaRRiS (see Supplementary appendix A). The free-play sessions were video recorded for the purpose of inter-rater reliability. P.L. attended the parent–child free-play sessions, set-up the sets of standardized toys (a nurturing set including a baby doll, clothing and accessories, and a Lego Duplo set including a farm house, people, animals and accessories) and camera, and then left the room for purposes of blinding. Parents were instructed to play with their child as they normally would. At the end of the parent–child interaction free-play session, parents were given a short questionnaire to complete (see table 1).

**Table 1:** Parental survey content summary

Topic	Notes/example items	Response
Experience of taking part in the free-play session	Four items, example item: ‘The instructions given to me at the beginning of the free-play session were easy to understand’.	Extent of agreement on a five-point Likert scale (strongly agree to strongly disagree)
Degree of comfort/discomfort with video recording	Single item asking if videoed, how comfortable they were being video recorded	Degree of discomfort experienced on five-point Likert scale (not at all uncomfortable to extremely uncomfortable)
Demographic details	Single or multiple birth Birth order Has child received diagnoses of developmental conditions? Who usually lives in the home? Age of parent when leaving full time education Languages other than English spoken in the home	Options: single, twin or multiple Options: first, second, third or more Yes/no; if yes, please specify Please list Open response Yes/no; if yes, please specify
Parental concerns	Worries about how your child talks and makes speech sounds Concerns about how your child understands what you say Has your child begun to combine words yet	No, yes, a little No, yes, a little Not yet, sometimes, often

### CCHN phone interviews

Phone interviews were conducted with the five CCHNs post parent–child interaction data collection. The interviews were conducted by a Speech and Language Therapy Masters student who was not known to the nurses in order to prevent confirmatory bias. The student used an interview guide which included questions such as: what was least/most useful about the workshop and training; what are the barriers/facilitators to using the rating scale; how would the observational rating scale fit into you work; how useful do you think the tool could be to help identify children who need additional help/services. Phone interviews with CCHN were recorded, with consent.

### Analysis

Both quantitative and qualitative analytical techniques were implemented. Inter-rater reliability was quantified for all 30 parent–child observations using the intra-class correlation coefficient (ICC), comparing the child health nurse scores (trained raters) to P.L.’s scores (expert-rater). In order to explore the CCHN’s views of using PaRRiS phone interview recordings were transcribed and thematically analyzed using a semantic approach, whereby the coding and themes represent the explicit content of the transcripts.^27^ Thematic analysis involves familiarization (transcribing and re-reading transcripts); coding (tagging a segment of data with a label); examining the codes and collating data to identify themes; reviewing themes (checking identified themes against the data to ensure they answer the research question); and defining and naming themes.^21^ Due to the complexities regarding targeting interventions according to family SES, we were also interested in examining associations between parent–child interaction quality and quantity and socioeconomic status. A *post hoc* analysis was conducted to explore the relationship between PaRRiS ratings and family socioeconomic status (based on postcode—similar to a zip code in other countries), using Spearman’s correlation.

## Results

### Parent–child dyad sample characteristics

The toddler participants were on average 28 months, 60% (18/30) were male, all were single births and no children were reported to have a developmental condition or hearing impairment (table 2). Two-thirds of parents attending the visit were mothers (66.7%), two were fathers (6.7%), five were both mother and father (16.7%) and three were other family members (10%: grandmother; foster mother; and a mother, father and older sister). Only one of the 28 parents who completed the survey was from a non-English speaking background (3.6%). Half of the families (*n* = 15) were from the most deprived 10% lower super-output areas in England based on the English Index of Multiple Deprivation (IMD).^28^ When reporting on their child’s language abilities, a quarter of parents (7/28) indicated they worry about how their child talks, 3.6% (1/28) said they were concerned about how their child understands and 18% (5/28) said their child has not begun to combine words yet. The average rating for parents on the PaRRiS was 3.0 (SD 0.8, range 1.0–4.0) based on 31 ratings (two nurses both rated one of the parent–child interactions). No parent–child interactions received a rating of 5.0 (very responsive).

**Table 2:** Caregiver and toddler sample characteristics

Characteristics	*N* = 28–30
Child age in months at visit, mean (SD)	28.0 (1.9)
Female, *%* (*n*)	40.0 (12)
*Caregiver at visit*	
Mother	66.7 (20)
Father	6.7 (2)
Mother and father	16.6 (5)
Grandparent/other	10.0 (3)
10% most deprived area, % (*n*)	50.0 (15)
Caregiver from non-English speaking background, % (*n*)	3.6 (1)
Worries about how child talks, % (*n*)	25.0 (7)
Concerns about how your child understands, % (*n*)	3.6 (1)
Child has not begun to combine words yet, % (*n*)	17.9 (5)

### RQ 1: reliability of CCHNs PaRRiS ratings

**Figure 1:**
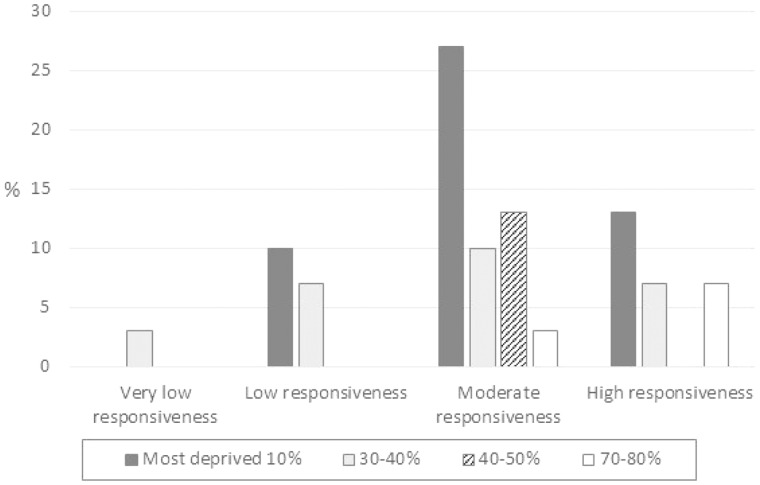
PaRRiS by English index of multiple deprivation deciles (*N* = 30)

Reliability between the first author (‘gold standard’) and child health nurses was calculated using ICC (absolute): 0.85; 95% CI: 0.67–0.93. An ICC of 0.75–1.00 is considered excellent reliability.^29^^,^^30^Figure 1 shows that families from the 10% and 30–40% most deprived areas were rated as very low to high responsiveness, while those in the 40–50% and 70–80% were rated moderate and high responsiveness. The association between social disadvantage (IMD) and responsiveness rating was *r_s_* = 0.08, *P* = 0.7.

### RQ 2: acceptability and feasibility of PaRRiS

Most parents indicated they were comfortable with the video recording, with 58.6% (17/29) of parents responding that they were quite comfortable and 37.9% (11/29) indicating that they were extremely comfortable being video recorded during the observational free-play session.

### CCHN evaluation interviews

Of the five CCHN participants, one was a health visitor with 13 years of experience. The other four were nursery nurses with experience ranging from 8 to 25 years. The three main themes to arise from the CCHN interviews were adequacy of training; acceptability and feasibility of using PaRRiS in practice; and benefits of PaRRiS. Themes and quotes are presented in table 3.

**Table 3:** Themes and quotes from CCHN participant interviews

Theme	Subtheme	Quotes
Adequacy of PaRRiS training	Suggestions for improvement	*Q1:* CCHN2: ‘It was really hard to understand until you started to do it. Um, so I felt the training was fine, but it wasn’t until I got into it that I really got what it was all about. I think it’s one of them tools that you’ve got to use’.
		*Q2:* CCHN4: ‘After reading through bits and pieces of it and talking to one of the health visitors who was involved in it, it was sort of a little bit easier to understand…but the practical side of it was pretty straightforward’.
Acceptability and feasibility of using PaRRiS in practice	Time as an initial concern	*Q3:* CCHN5: ‘it sort of comes out of the assessment if that makes sense…so it was an extra 10 min on an already long assessment…so that was probably the main thing I would say, and I guess, no I think using the tool was fine actually’.
		*Q4:* CCHN2: ‘It did add time, but not hugely, I mean we factored that into the time we allocated, so we weren’t rushing’.
	Applicability to a home context	*Q5:* CCHN5: ‘There’s too many distractions, they’re up and down for other things, you know, you can’t clear the living room to just put suitable, suitable toys down there, there’s all sorts of random stuff’.
	Effect of observation on parental behaviour	*Q6:* CCHN4: ‘Actually on one of the ones I observed, the parent was really nervous, you know, you could see she was nervous and I think that made her do things that she wouldn’t necessarily do, in a natural environment…so I think if the video recorder’s not there it’s a whole different idea’.
Value add of PaRRiS to practice	Identifying what parents are doing and where they could benefit from support	*Q7:* CCHN1: ‘I think practitioners could be trained to be guided on…getting children to lead play and things like that cause I think you do, you know we look to see how responsive parents are and we use that saying don’t we, ‘responsive parenting’, I think actually by really pinpointing it down to a scale will make it really clear how responsive they are and in what way we mean they’re responsive to their child, because I think we use that term quite widely without being really clear what we’re seeing really’.
		*Q8:* CCHN3: ‘It’s helpful…to make us aware that, well ok, the parents aren’t doing what they say they’re doing; therefore, we need to work them so they can develop their child’s skills’.
	Assisting with the referral process	*Q9:* CCHN2: ‘I think sometimes we refer to speech and language a bit too soon. And by the time they get there, because of waiting lists, by the time they get there, often things have improved. And I think this might just give us, a little bit more um, a bit more understanding I think of where we may need to go with it’.

CCHN, community child health nurse (health visitor or nursery nurse; and numbers indicate the specific participant); Q, quote.

#### Adequacy of PaRRiS training

The practical element of the training was viewed as most important and doing more example videos in training, rather than in their own time, was considered beneficial. CCHNs suggested watching and rating all example clips in the workshop rather than taking them away would be of benefit (see table 3: Q1). While CCHNs stated training was adequate, some also highlighted a need for their knowledge gaps/needs to be identified prior to training to ensure the information provided in the session was relevant and communicated meaningfully (Q2).

#### Acceptability and feasibility of using PaRRiS in practice

The added amount of time that it would take to include observations of parent–child interaction as part of routine checks was of initial concern to participating CCHNs pre-data collection (Q3), but they did acknowledge that in practice the time added was not too great (Q4).

All observations were carried out at children’s centre or healthcare clinics, but in practice, visits can often be conducted in the home, so implementation may be quite different in a home setting (Q5). In addition, one CCHN raised the issue of whether or not parents behave as they normally would when videoed, but also acknowledged that as we were video recording for purposes of reliability, PaRRiS without video might not impact parental behaviour (Q6).

#### Value add of PaRRiS to practice

CCHNs reflected on the potential benefits of using PaRRiS in their daily practice, as well as the potential of the tool to be used more broadly by practitioners working with children in the early years (Q7 and Q8). CCHNs expressed the added value of the PaRRiS tool as providing information not only just on the child’s development but also information on the parent–child interaction which may assist in preventing over-referrals (Q9).

## Discussion

Findings from this study demonstrate that CCHNs (with a range of years of experience and qualifications) can be trained to reliably measure parental responsiveness using the PaRRiS tool during a brief 5-min free-play observation. Qualitative evidence from the trained CCHNs suggest it is an acceptable and feasible supplementary tool for use in everyday practice to provide additional information about the parent’s quality of responsiveness if there is concern regarding the child’s language development and/or parent–child interaction. Given the recognition by practitioners across Europe of the importance of parent–child interaction, and that parental responsiveness is an important factor for child language development in other European cultures,^19^^,^^31^^,^^32^ there is potential for the adaptation and use of PaRRiS beyond the UK.

The demonstrated acceptability, feasibility and reliability of PaRRiS suggests further development and evaluation work is worthwhile. To develop the tool to a point where it would be ready for widespread adoption in practice, additional steps are required. First, development of a training model which can be delivered at scale, allowing practitioners to develop their observation skills to a measurable criterion of reliability is required. Second, an intervention study is required to test our hypothesis that PaRRiS can identify parent–child dyads most likely to require intervention, given PaRRiS has been shown to strongly predict child language outcomes (see Hudson et al.^26^). This will involve comparing outcomes for parents identified using PaRRiS to those using clinician judgement informed by current universal assessment practices and measuring both parent and child outcomes. Third, the cost-effectiveness of the use of PaRRiS by CCHNs to identify those most likely to benefit from parent–child interaction interventions should be estimated.

Our secondary findings support those who express concerns regarding the targeting of parent–child interaction interventions based solely on SES.^33^ In this sample of parent–child dyads, there was no evidence of a relationship between social disadvantage and parental responsiveness ratings. Clearly this requires further testing in a larger sample, but it would suggest that preventative intervention models must acknowledge the wide variety of language environments which exist in families living with social disadvantage or risk providing either the wrong kind or unnecessary support.

Despite challenges to the claims that socially disadvantaged parents provide less-rich language environments the social gradient in child language outcomes has been replicated across a number of cohorts.^34^ Rather than focussing *only* on parental behaviours as a driver of this gradient we suggest that researchers, policy-makers and practitioners must acknowledge the broader social and structural inequalities which make provision of optimal home-learning environments challenging.^24^ Perhaps targeted selective approaches for socially disadvantage families should focus on social and structural barriers and enablers to optimal home learning rather than, or in addition to, parent–child interaction interventions.

Furthermore, PaRRiS could inform a *targeted indicated* approach which considers both child language and parental-responsiveness as factors to indicate the need for intervention.^23^ This could be in the form of a two-stage screening process, whereby a concern for a child’s language development and/or parent–child interaction, could trigger the CCHN to conduct a 5-min observation using PaRRiS. The decision to offer parent–child interaction intervention would then be informed by this rating. Furthermore, the model of intervention provided should be tailored with respect to the family’s resources and ability to access the intervention^35^^,^^36^ with families with greater resources receiving ‘light touch’ advice and those with less receiving more extended coaching.^37^

### Strengths and limitations

A strength of the study is that it is a community-based sample, including families from a range of socioeconomic backgrounds, including a high proportion of families with lower SES as well as a range of primary caregivers (i.e. not only mothers). As recruitment was restricted to families attending centres for their reviews, not those having home visits, the sample is representative only of those families who are more likely to attend a centre for their reviews (i.e. ‘harder-to-reach’ families are likely to be under-represented). Recruitment rate of families was high (30/31) and care was taken to minimize potential biases through blinding and independence of the qualitative interviewer. Recruitment of only five child health nurses, slow recruitment of families and constraints on study time and resources resulted in a relatively small sample size. The fact that some of the CCHNs completed only a few observations is a further limitation.

## Conclusions

The demonstrated acceptability, feasibility and reliability of PaRRiS suggests that further development and evaluation work is worthwhile. PaRRiS has the potential to be incorporated into existing universal health services to provide CCHNs with an additional tool for identifying families most in need of parent–child interaction interventions and hence promote robust child language development in the crucial early years.

## Supplementary data

Supplementary data are available at *EURPUB* online.
